# *APOE* Allele Frequency in Southern Greece: Exploring the Role of Geographical Gradient in the Greek Population

**DOI:** 10.3390/geriatrics8010001

**Published:** 2022-12-20

**Authors:** Vasiliki Papastefanopoulou, Evangelia Stanitsa, Christos Koros, Aimilios Simoudis, Chryseis Florou-Hatziyiannidou, Ion Beratis, Roubina Antonelou, Nikolaos Andronas, Panagiota Voskou, Efthalia Angelopoulou, John D. Papatriantafyllou, Leonidas Stefanis, Christos Kroupis, Sokratis G. Papageorgiou

**Affiliations:** 1 Department of Clinical Biochemistry and Molecular Diagnostics, Attikon University General Hospital, Medical School, National and Kapodistrian University of Athens, 12462 Chaidari, Greece; 2 Cognitive Disorders/Dementia Unit, 2nd Department of Neurology, Attikon University General Hospital, Medical School, National and Kapodistrian University of Athens, 12462 Chaidari, Greece; 3 1st Department of Neurology, Aiginiteio University Hospital, National and Kapodistrian University of Athens, 11528 Athens, Greece; 4 Psychology Department, American College of Greece, Deree, 15342 Agia Paraskevi, Greece; 5 Third Age “IASIS”, Athens and Memory Clinic, Medical Center of Athens, 11636 Athens, Greece

**Keywords:** Alzheimer’s disease, mild cognitive impairment, Apolipoprotein E, Greek population

## Abstract

Background: the apolipoprotein e4 allele (*APOE4*) constitutes an established genetic risk factor for Alzheimer’s Disease Dementia (ADD). We aimed to explore the frequency of the *APOE* isoforms in the Greek population of Southern Greece. Methods: peripheral blood from 175 Greek AD patients, 113 with mild cognitive impairment (MCI), and 75 healthy individuals. DNA isolation was performed with a High Pure PCR Template Kit (Roche), followed by amplification with a real-time qPCR kit (TIB MolBiol) in Roche’s Light Cycler PCR platform. Results: *APOE4* allele frequency was 20.57% in the ADD group, 17.69% in the MCI group, and 6.67% in the control group. *APOE3/3* homozygosity was the most common genotype, while the frequency of *APOE4/4* homozygosity was higher in the AD group (8.60%). *APOE4* carrier status was associated with higher odds for ADD and MCI (OR: 4.49, 95% CI: [1.90–10.61] and OR: 3.82, 95% CI: [1.59–9.17], respectively). Conclusion: this study examines the *APOE* isoforms and is the first to report a higher *APOE* frequency in MCI compared with healthy controls in southern Greece. Importantly, we report the occurrence of the *APOE4* allele, related to ADD, as amongst the lowest globally reported, even within the nation, thus enhancing the theory of ethnicity and latitude contribution.

## 1. Introduction

Alzheimer’s disease (AD) is the most common neurodegenerative disorder with multifactorial etiology. One of the most important genetic factors associated with AD is the apolipoprotein E gene (*APOE*) [1]. The *APOE* gene encodes a 299 amino-acid protein, and it has three common isoforms known as *APOE2, APOE3,* and *APOE4*. Single amino acid differences between these three isoforms affect their structure and activity. *APOE* plays a key role in neuronal maintenance and repair as it is critically involved in lipid transport and metabolism in the periphery and the brain [2]. The *APOE4* allele is a well-documented genetic risk factor [3,4] for the development of AD [5], while the *APOE3* allele seems to be neutral, and *APOE2* is considered to be protective [6,7].

With regards to the amyloid-β (Aβ) hypothesis for AD development [8,9], *APOE4* can regulate amyloid precursor protein (APP) processing, Aβ production, and potentially Aβ aggregation via several mechanisms including the LRP (lipoprotein receptor-related protein) pathway [1,10].

*APOE4* occurs in up to 50% of patients with Alzheimer’s Disease Dementia (ADD) [11] and increases the risk for sporadic and familial, early and late-onset of the disease [12,13,14,15]. In particular, *APOE4* homozygosity may increase 15-fold the possibility of developing AD at an earlier age [16,17,18]. Even one copy of the *APOE4* allele increases the risk of AD compared with individuals with *APOE3* homozygosity [19]. The presence of the *APOE4* allele renders women more susceptible to developing ADD than men [20] and at younger ages [21], although there is also conflicting evidence [22,23]. When it comes to homozygosity, *APOE4* carriers showed an augmented risk in AD compared with *APOE3* homozygotes for both men and women [23].

Meanwhile, the *APOE4* genotype also correlates with mild cognitive impairment (MCI), which is often seen as a precursor to ADD [24,25,26]. *APOE4* homozygosity raises the risk for ADD by up to 30% in patients with MCI [27], a stage between healthy aging and dementia, taking hold of 16–20% of individuals above the age of 60 [28]. MCI patients carrying at least one *APOE4* allele have lower performance in memory tests [29,30] and lower hippocampal volume compared with non-carriers [31]. These changes occur in *APOE4* carriers 5–15 years prior to symptom onset compared with non-carriers [17]. Studies showed that, as in ADD, in MCI, *APOE4* implicates amyloid accumulation, lowers CSF Aβ, and increases CSF tau levels [32]. Furthermore, men and women with MCI carrying *APOE4* perform differently on memory tests, suggesting that sex differences might affect the progression toward ADD [21,33].

According to previous studies, *APOE* allele frequency seems to depend on ethnicity and geographical latitude indicating a decreased rate from northern to southern Europe [34,35,36]. In Greece, *APOE* allele frequencies have been previously investigated in healthy individuals, refs. [37,38,39] patients with dementia [40], or MCI [41]. The aim of this study was to explore the *APOE* allele frequencies in healthy individuals and patients with MCI and ADD in the southern part of Greece. To the best of our knowledge, this is the first study to explore the APOE frequencies in patients with MCI in this part of the country. Thus, this study completes the overall recording of the *APOE* genetic analysis in the Greek population among healthy individuals and patients with ADD and MCI.

## 2. Materials and Methods

### 2.1. Participants

In the current study, *n* = 113 patients with MCI, *n* = 175 patients with ADD, and *n* = 75 healthy individuals were recruited. MCI and ADD patients were referred to the Attikon University General Hospital Cognitive Disorders/Dementia Unit of the second Department of Neurology at the National and Kapodistrian University of Athens for a thorough clinical evaluation. All participants of the current study were residents of Southern Greece. The recruitment period started in 2015 and ended in 2019. All patients underwent a neurological evaluation and a cognitive screening assessment, including the Mini-Mental State Examination tests (MMSE) [42] and frontal assessment battery (FAB) [43]. Medical and family history were collected and verified by an informant. Brain MRI or CT scan and blood tests were also required for the diagnostic process. The diagnosis of MCI was made in accordance with the Petersen and Morris criteria [44], along with a score of 0.5 on the Clinical Dementia Rating Scale (CDR) [45], while the diagnosis of ADD was made according to the McKhann criteria [46] along with a CDR score of 1. All diagnoses were made by the same behavioral neurologist [SGP] with expertise in dementia and cognitive disorders. 

The control group (henceforth CTRL) of the current study consisted of healthy individuals accompanying the patients to their consecutive visits to the unit as informants. Individuals above the age of 40, with no reported subjective cognitive decline, were recruited. Individuals with subjective cognitive decline were carefully excluded from the current study.

### 2.2. Materials

Blood was collected in EDTA-containing tubes from well-ascertained MCI and ADD patients and healthy participants recruited from the southern part of Greece. After collection, these samples were centrifuged within 4 h to obtain a buffy coat of white cells. The genomic DNA was extracted from 200 μL of buffy coat using the High Pure PCR Template Kit (Roche, Penzberg, Germany). For the amplification of the *APOE* gene, 30 ng of genomic DNA was amplified using a real-time qPCR kit (TIB MolBiol, Berlin, Germany) in the Light Cycler PCR platform (Roche) [47]. Ambiguous or positive samples for the *APOE4* allele were confirmed by conventional PCR followed by HhaI restriction digestion and analysis in ethidium bromide-stained 4% high-resolution agarose gels [48].

### 2.3. Ethical Considerations

This study was in accordance with the latest Helsinki Declaration [49]. Also, it has been approved by the Bioethics Committee of the Attikon General University Hospital (987/9–9-2015). Informed consent was obtained from all the participants. It was highlighted to them that their participation would be voluntary and that they had the right to withdraw at any time, as well as that their personal data would remain confidential and would only be used for research purposes, according to the General Data Protection Regulation (EC 2016/676). 

### 2.4. Statistical Analysis

Statistical analysis was performed using the SPSS statistics software version 21 (IBM Corp. Armonk, NY, USA). The statistical significance level was set at *p* < 0.05. For the demographic characteristics, descriptive statistics were performed (means and standard deviations for continuous variables and frequencies for categorical variables). Continuous variables (age, education years, MMSE, and FAB total scores) were not normally distributed; therefore, in order to examine if the three diagnostic groups—including the cognitively intact control individuals (CTRL), patients with MCI, and patients with ADD—were comparable based on their characteristics, the Kruskal–Wallis and Mann–Whitney U tests were used. Chi-squares of independence were used to explore the associations between categorical variables such as sex. Binary logistic regression models were applied to assess the associations between diagnostic groups and *APOE* alleles, using age, and education years as covariates, and sex stratification of the results. The same analysis was used for genotypes.

## 3. Results

### 3.1. Participants’ Characteristics 

Demographic characteristics (age in years, education in years, and sex) and cognitive measures (MMSE and FAB) are presented in Table 1. The application of Kruskal–Wallis indicated that the CTRL group was significantly younger than patients with MCI and patients with ADD (*p* < 0.001). In addition, patients with ADD had significantly fewer education years compared with patients with MCI and CTRL (*p* = < 0.001). MCI patients had more years of education in comparison with patients with AD (*p* = 0.024) according to the Mann–Whitney U that was applied. The general cognitive ability of the CTRL group, measured with the MMSE score, was significantly higher than the group of patients with MCI and the group of patients with AD, while the group of patients with MCI had a substantially higher MMSE score than the group of patients with AD (*p* < 0.001). The CTRL group had a significantly higher FAB score than both MCI and ADD groups, while the MCI group had a significantly higher score than patients with ADD (*p* < 0.001). Application of the chi-square test for sex differences between each group indicated that women were significantly more than men in the CTRL group χ^2^ (1, *n* = 75) = 8.33 (*p* = 0.004), but there were no significant sex differences in the MCI group χ^2^ (1, *n* = 113) = 0.43 (*p* = 0.510) nor the ADD group χ^2^ (1, *n* = 175) = 0.46 (*p* < 0.496). 

### 3.2. APOE Allele and Genotype Frequencies 

In Table 2, *APOE* allele frequencies are presented. Allele frequency was determined by counting how many copies of each allele appeared in our sample divided by the total number of gene copies.

According to the results reported in Table 2, the frequency of the *APOE4* allele was 20.57% in the ADD group and 17.69% in the MCI group, while the lowest frequency was indicated in the CTRL group (6.67%). The *APOE2* allele frequencies were generally low. Application of the chi-square test for independence indicated that the *APOE2* frequencies of the CTRL did not significantly differ from ADD patients’ χ^2^ (1, *n* = 500) = 0.04 (*p* = 0.834) nor from the MCI patients’ χ^2^ (1, *n* = 376) = 0.28, (*p* = 0.596), while *APOE2* frequencies of the patients with MCI did not significantly differ from ADD patients’ χ^2^ (1, *n* = 576) = 0.18 (*p* = 0.671). Application of the chi-square test for independence indicated that the *APOE3* frequencies of the CTRL significantly differ from ADD patients’ χ^2^ (1, *n* = 500) = 13.39 (*p* < 0.001) and from the patients with MCI χ^2^ (1, *n* = 376) = 9.27, (*p* = 0.002), while *APOE3* frequencies of the patients with MCI did not significantly differ from ADD patients’ χ^2^ (1, *n* = 576) = 0.36 (*p* = 0.550). Application of the chi-square test for independence indicated that the *APOE4* frequencies of the CTRL significantly differ from ADD patients’ χ^2^ (1, *n* = 500) = 14.81 (*p* < 0.001) and from the patients with MCI χ^2^ (1, *n* = 376) = 9.52, (*p* = 0.002), while *APOE4* frequencies of the patients with MCI did not significantly differ from ADD patients’ χ^2^ (1, *n* = 576) = 0.72 (*p* = 0.395). 

As presented in Table 3, the frequency of the *APOE4/4* homozygosity was higher in the ADD group (8.60%) than in the MCI group (2.70%). *APOE3/4* frequency was highest in the MCI group (29.20%), high in the ADD group (24.00%), and lower in the CTRL group (9.30%). *APOE2* homozygosity was not found in the current study sample, while *APOE2* heterozygosity showed low frequency in all the groups but was slightly higher in the control group. *APOE3/3* homozygosity was the most frequent genotype in all three groups. Application of the chi-square test for independence did indicate that the *APOE* genotypes of the CTRL significantly differed from ADD patients’ χ^2^ (4, *n* = 250) = 16.77 (*p* = 0.002) and MCI patients’ χ^2^ (4, *n* = 188) = 12.86 (*p* = 0.012). However, the difference between MCI and AD patients’ *APOE* genotypes was not significant: χ^2^ (4, *n* = 288) = 6.14 (*p* = 0.189).

By the exploration of the presence of *APOE2* and *APOE4* in both homozygotes/heterozygotes groups and the association of *APOE4* with clinical diagnosis, it was indicated that the presence of *APOE2* in both homozygotes and heterozygotes group was generally low in all the groups of participants, with the highest frequency in MCI group (8.80%). *APOE4* presence was equally high in the ADD patients (32.60%) and the MCI patients (32.70%), while a low frequency was indicated in the CTRL group (12.00%). The application of the chi-square test for independence showed a significant association between the presence of at least one *APOE4* allele and the clinical diagnosis of ADD (*p* = 0.001) as well as the *APOE4* allele and the clinical diagnosis of MCI (*p* = 0.001). 

### 3.3. Odd Ratios for ADD and MCI Carriers of the APOE4 

A logistic regression analysis was performed between each patient group and the CTRL group, adjusted for age because the AD and MCI patients were significantly older than the CTRL participants. The sample was stratified by sex. As stated in Table 4, the likelihood of ADD in *APOE4* carriers was 4.49 times higher than the likelihood of non-carriers for the specific allele (OR = 4.49, 95% CI = [1.90–10.61]). In particular, the likelihood of ADD in female carriers of the *APOE4* were 5.18 times higher than the likelihood for individuals that are non-carriers (OR = 5.18, 95% CI = [1.71–15.64]). However, the likelihood of ADD for male *APOE4* carriers were not significantly different compared with non-carriers. In addition, the likelihood of MCI for *APOE4* carriers were 3.82 times higher than for non-carriers (OR = 3.82, 95% CI = [1.59–9.17]). In particular, the likelihood of MCI for female carriers of the *APOE4* were 4.47 times higher than the likelihood for non-carriers (OR = 4.47, 95% CI = [1.42–14.04]). The likelihood of MCI for male carriers were not significantly different compared with non-carriers.

A logistic regression analysis was performed between the MCI and the ADD group, adjusted for age and education because the ADD patients were significantly older and less educated than the MCI participants. The sample was stratified by sex. When used as a reference category for the diagnosis of MCI, the likelihood for ADD for carriers of *APOE4* was not significantly higher than for non-carriers (Table 4).

Logistic regression was performed to assess the likelihood of the CTRL group according to their genotype, adjusted for age, and stratified by sex, for ADD. The odds for each genotype (*E2*/*E2*, *E2*/*E3*, *E2*/*E4*, *E3*/*E4*, *E4*/*E4*) were compared with the *E3*/*E3* genotype, which is the most frequent type. As presented in Table 5, when using the control group as a reference point, the odds for individuals with the *E3*/*E4* genotype for ADD were 5.03 times higher than the odds for individuals with the *E3*/*E3* genotype (OR = 5.03, 95% CI = [1.85–13.68]). In particular, the odds for females with the *E3*/*E4* genotype for ADD were 5.94 times higher than the odds for females with the *E3*/*E3* genotype (OR = 5.94, 95% CI = [1.71–20.63]), while the odds for males with the *E3*/*E4* genotype for ADD were not significantly higher than the males with *E3*/*E3*. In addition, the odds of individuals with the *E4*/*E4* genotype for ADD were 11.04 times higher than the odds for individuals with the *E3*/*E3* genotype (OR = 11.04, 95% CI = [1.31–93.18]). No other genotype indicated statistically significant odds. 

In the same vein, logistic regression was performed to assess the odds for ADD according to their genotype by using the diagnosis of MCI as a reference category, adjusted for age and education, and stratified by sex. The odds for each genotype (*E2*/*E2*, *E2*/*E3*, *E2*/*E4*, *E3*/*E4*, *E4*/*E4*) were compared with the *E3*/*E3* genotype, which is the most frequent type. However, there was not any statistically significant likelihood (Table 5).

## 4. Discussion

The aim of our study was to investigate the *APOE* frequencies in healthy individuals, patients with MCI, and patients with ADD in a Southern Greece population. According to the results, *APOE4* allele frequency was 6.67% in the CTRL group, 17.69% in the MCI group, and 20.57% in the ADD group. *APOE2* allele frequency was 3.3% in the CTRL group, 4.4% in patients with MCI, and 3.7% in patients with ADD. *APOE3* allele frequency, as expected, was the highest, with 90% in the CTRL group, 77.9% in the MCI group, and 75.7% in the ADD group. The *E4*/*E3* genotype was high in patients with MCI (29.20%) and the ADD group (24.00%). *E4* homozygosity was 8.60% in patients with ADD, 2.70% in patients with MCI, and 1.30% in the CTRL group. *E3*/*E3* indicated the highest prevalence in all groups. These results are in accordance with previous studies exploring the increased risk for ADD, linked to the occurrence of *APOE4* [12,13,14,15] and the homozygosity’s higher odds for ADD [16,17,18]. To the best of our knowledge, this is the first study exploring *APOE* frequency in patients with MCI in southern Greece. 

Healthy female carriers exhibited 5.18 times significantly higher odds for ADD and 4.47 times significantly higher odds for MCI than non-carriers, while the odds for male carriers did not significantly differ from non-carriers. There is conflicting evidence regarding whether female carriers are at greater risk for ADD than men, as suggested by a recent meta-analysis exploring how sex and *APOE* genotype affects the risks for MCI and ADD. They concluded that men and women have nearly the same odds of developing ADD from age 55 to 85 years, but women have an increased risk at younger ages (from 65 to 75) [21]. On the other hand, another meta-analysis showed a four-fold risk in heterozygous women in their sixties and a twelve-fold risk in homozygous women, but not as high as for men [19]. The results of the current study support that the impact of *APOE4* for ADD is far more pronounced in women than men, indicating a possible sex–APOE interaction [20,50,51]. Regarding patients with MCI, although the association between *APOE4* and sex has not been established [21,50], according to Nue [21], women at younger ages had an increased risk for MCI compared with men, but not at older ages. In addition, Altmann found an interaction between *APOE4* and the female sex for MCI and/or AD. 

Previous studies in Greece, including healthy individuals, indicated that *APOE4* allele frequency ranged from 6.5% to 13.6%, *APOE2* allele frequency ranged from 4.1% to 8.1%, and *APOE3* allele frequency ranged from 79.3% to 88.2% [37,38,39,40,41,52,53]. Interestingly, the *APOE4* frequency in northern Greece for the healthy cognitive cohort was 13.1%, while our study in southern Greece indicated a lower frequency of 6.67%. It seems that there is a gradient of *APOE4* allele distribution among populations, with higher *APOE4* allele prevalence in northern parts of Greece and lower allele prevalence in central and southern parts of the country. This is consistent with studies in the European population which have revealed a gradual decrease in *APOE4* allele frequency from the Nordic to the Mediterranean countries. APOE polymorphism follows a latitudinal pattern [54], showing a different allele frequency of APOE by geographic region, while significant differences have also been observed between Caucasian, Asian (Chinese and Japanese), and Black races [37,55]. AD patients in southern European/Mediterranean communities have been estimated to display significantly lower *APOE4* carrier status (43%) compared with those in northern Europe (64%) [34]. Besides Greece, this descending southwards *APOE4* trend is also observed between regions of the same country, as shown for the United Kingdom, France, Spain, and Italy (please see Supplementary Tables S1 and S2). Moreover, herein in the study, the *APOE4* allele frequency in the control group was low (6.67%) and aligned with other studies in southern Europe, such as Madrid of Spain (6.10%), Sardinia (5.20%) and Sicily (5.8%). Ref. [56] The pattern of low *APOE4* frequencies could be explained by a natural protecting feature, typical to Mediterranean populations, known as the Mediterranean paradox. The genetic component in this theory is that genetic variants accounted as risk factors for several diseases may have a diverged incidence at the population level [57]. Although the *APOE4* allele is related to increased total and low-density lipoprotein (LDL) levels, Mediterranean environmental and dietary factors regulate cholesterol metabolism [58,59]. In the same vein, the lower prevalence of the *APOE4* allele in southern European populations and the Mediterranean diet, which is low in fat intake, have been associated with a more beneficial lipid profile and lower cardiovascular mortality rates compared with northern Europeans [35,60,61,62]. Nevertheless, the latitudinal pattern that the *APOE4* seems to follow from north to south may also be explained due to environmental causes. The cold temperatures of high-latitude northern Europe demand higher cholesterol levels and, therefore, accelerated metabolic rates [59].

Another potential interpretation is related to the history of the European continent and especially the massive trends of migration and admixture events [63]. Consequently, populations along the Mediterranean coast share a genetic heritage that surpasses ethnicity, including the Southern Italian population, Greeks, and Spanish. This similar genetic background traces back to prehistoric times as the result of multiple migration waves during the Neolithic and the Bronze Age, with peaks during the imperial period of Rome and Ancient Greece [64]. From the 8th to 5th century BC, the imperialistic tendency of Greeks created the Magna Graecia, thus naming the coastal line area of the Tyrrhenian Sea. This extended the Greek maritime route to the south and coastal Italy and acted as a bridge for population admixture, and hence, genetic parallelism [65]. Relatedly, Stamatoyannopoulos clarified an important aspect, having Greeks, Sicilians, and Italians interact genetically stronger than any other population of the Mediterranean basin, such as Basques, Andalusians, and French [66]. Also, a study by Di Gaetano explained that the Sicilian genetic system lacks its original diversity at a portion of 60% because of Greek admixtures that happened in antiquity [67]. To explore this gene flow across the Mediterranean and its homogeneity, studies using SNP panels found mitochondrial and chromosomal similarities among these populations that meddled in human evolutionary history [68,69,70].

Furthermore, other genetic factors could modify the *APOE4* allele-related toxicity and subsequent AD risk. One of them is the KLOTHO gene, a factor that affects the neurodegeneration pathways by diminishing the harmful action of the *APOE4* allele, and by delaying the mechanisms related to aging [71]. A study proved that KL-VS heterozygotes in the KLOTHO gene and *APOE4* carriers did not demonstrate a higher Aβ burden than *APOE4* non-carriers [72,73]. Likewise, Christchurch *APOE* gene mutation (R136S) also seems to play a subsidiary role in the *APOE*’s clearance dexterity; it operates as a protective layer for neurons and cells to prevent *APOE* from binding with other molecules and *p*-tau from accumulating [74]. The Christchurch study underlines the profound importance of *APOE* in brain cascading that leads to AD. Additionally, mitochondrial dysregulations have an essential impact on cellular activity, leading to the pathogenesis of several neurodegenerative diseases. A dysfunction in the translocase of the outer mitochondrial membrane (TOMM 40) is believed to be involved in the pathophysiology of AD. Interestingly, the TOMM 40 gene is known to be downregulated in the brain and blood of patients with AD. Studies suggest that TOMM 40 implication to AD onset synergistically lies with the *APOE* gene by relating to similar genome regions [75,76]. Hence, future studies investigating the frequencies of TOMM40 SNPs across different geographical latitudes including northern and southern regions in Greece would be of particular interest, especially in combination with the frequencies of *APOE* alleles. Overall, all these findings are promising for designing potential biomarkers for early disease detection and assessment.

## 5. Conclusions

To conclude, in the present study, we investigated *APOE* allele frequencies in Southern Greece, reporting—to the best of our knowledge for the first time—the *APOE* allele frequencies for patients with MCI. Our results confirm that the *APOE4* allele frequency in Greece remains amongst the lowest globally reported rates, providing a better understanding of the genetic background between northern and southern regions, and enhancing the theory of ethnicity and latitude contribution to genetic diversity. In a world orbited on clinical trial stratification and genetic breakthrough, the identification of the genetic distribution of *APOE4* variants within ethnicities and latitudes may aid in elucidating its role in leading AD pathogenesis and contribute to the development of more targeted therapeutic approaches.

## Figures and Tables

**Table 1:** Demographic Characteristics of the participants of the study.

Group	CTRL (*n* = 75)	MCI (*n* = 113)	ADD (*n* = 175)		
				Kruskal Wallis	Mann-Whitney U Tests
				*p*-Value	
Age (years) *M ± SD*	62.63 ± 11.13	71.73 ± 7.94	72.45 ± 7.99	<0.001 *	CTRL < MCI **, CTRL < ADD **
Sex (men/women %)	33.30/66.70	53.10/46.90	47.40/52.60	-	-
Education *M ± SD* (years)	13.80 ± 1.09	11.28 ± 4.18	8.27 ± 3.82	<0.001 *	ADD < MCI *,
MMSE *M ± SD*	29.02 ± 1.06	26.66 ± 2.64	19.74 ± 6.04	<0.001 **	CTRL > ADD *
FAB *M ±SD*	16.60 ± 1.41	13.30 ± 2.85	9.54 ± 4.07	<0.001 **	CTRL > ADD **, MCI > ADD **

Note: * *p* < 0.05, ** *p* < 0.001, MMSE: Mini-Mental State Examination total score, FAB: frontal assessment battery total score, CTRL: control individuals, ANOVA: analysis of variance, SD: standard deviation.

**Table 2:** *APOE* allele frequencies in CTRL group, patients with MCI, and patients with ADD.

Group	Participants	Alleles, *n*	*APOE2* Allele, %	*APOE3* Allele, %	*APOE4* Allele, %
CTRL	75	150	3.33	90.00	6.67
MCI	113	226	4.42	77.87	17.69
ADD	175	350	3.71	75.71	20.57
Comparisons χ^2^ (*p*-value)					
CTRL vs. ADD			0.04 (0.834)	13.39 (<0.001 **)	14.81 (<0.001 **)
CTRL vs. MCI			0.28 (0.596)	9.27 (0.002 *)	9.52 (0.002 *)
MCI vs. ADD			0.18 (0.671)	0.36 (0.550)	0.72 (0.395)

Note: * *p* < 0.05, ** *p* < 0.001.

**Table 3:** *APOE* absolute and relative frequencies of genotypes in CTRL group, patients with MCI, and patients with ADD.

Group	*APOE* Genotypes, *n* (%)					
	*APOE2/E2*	*APOE2/E3*	*APOE2/E4*	*APOE3/E3*	*APOE3/E4*	*APOE4/E4*
CTRL	0(0.00)	4(5.30)	1(1.30)	62(82.70)	7(9.30)	1(1.30)
MCI	0(0.00)	9(8.00)	1(0.90)	67(59.30)	33(29.20)	3(2.70)
ADD	0(0.00)	13(7.40)	0(0.00)	105(60.00)	42(24.00)	15(8.60)
Comparisons χ^2^ (*p*-value)						
CTRL vs. ADD		16.77, (0.002 *)				
CTRL vs. MCI		12.86, (0.012 *)				
MCI vs. ADD		6.14, (0.189)				

Note: * *p* < 0.05.

**Table 4:** The likelihood ratios for ADD (ADD vs. CTRL), MCI (MCI vs. CTRL), and AD (ADD vs. MCI according to *APOE4* presence adjusted for age and stratified by sex.

*APOE4 +/−*	*p*	OR	95% CI
ADD vs. CTRL	0.001 **	4.49	1.90–10.61
ADD men	0.063	3.75	0.93–15.09
ADD women	0.004 *	5.18	1.71–15.64
MCI vs. CTRL	0.003 *	3.82	1.59–9.17
MCI men	0.104	3.20	0.79–13.02
MCI women	0.010 *	4.47	1.42–14.04
ADD vs. MCI	0.410	1.55	0.55–4.37
ADD men	0.892	1.12	0.22–5.60
ADD women	0.212	3.02	0.53–17.13

Note: * *p* < 0.05, ** *p* < 0.001.

**Table 5:** The odds for ADD (ADD vs. CTRL) according to genotype, adjusted for age, and stratified by sex; and the odds for ADD (ADD vs. MCI) according to genotype adjusted for age, education, and stratified by sex.

	*Ε2/2*	*Ε2/3*	*Ε2/4*	*Ε3/4*	*Ε4/4*
ADD vs. CTRL					
*p*-value	-	0.199	-	0.002 *	0.027 *
OR	-	2.33	-	5.03	11.04
95% CI	-	0.64–8.46	-	1.85–13.68	1.31–93.18
Men					
*p*-value	-	0.669	-	0.095	-
OR	-	1.63	-	4.37	-
95% CI	-	0.17–15.16	-	0.77–24.68	-
Women					
*p*-value	-	0.148	-	0.005 *	0.130
OR	-	3.39	-	5.94	5.93
95% CI	-	0.65–1.07	-	1.71–20.63	0.59–59.31
ADD vs. MCI					
*p*-value	-	0.576	-	0.422	0.587
OR	-	1.76	-	1.58	1.90
95% CI	-	0.24–12.72		0.52–4.83	0.19–19.07
Men					
*p*-value	-	0.607	-	0.994	-
OR	-	2.15	-	1.01	-
95% CI	-	0.12–40.19		0.17–5.84	-
Women					
*p*-value	-	-	-	0.149	0.814
OR	-	-	-	5.21	1.34
95% CI	-	-	-	0.56–48.88	0.12–15.43

Note: * *p* < 0.05.

## Data Availability

Available upon request to the corresponding author.

## Supplementary Materials

The following supporting information can be downloaded at: https://www.mdpi.com/article/10.3390/geriatrics8010001/s1, refs. [77,78,79,80], Table S1: *APOE4* allele frequency in different healthy ethnic populations; Table S2: *APOE4* allele frequency in healthy individuals in various parts of Greece.
