# Supplementary material for: APOE Allele Frequency in Southern Greece: Exploring the Role of Geographical Gradient in the Greek Population
Source: Geriatrics (Basel). 2022 Dec 20;8(1):1. doi: 10.3390/geriatrics8010001 (PMC9844375; doi:10.3390/geriatrics8010001)
Supplement: Supplementary file 1 [file geriatrics-08-00001-s001.zip › geriatrics-2078202-supplementary.pdf]

Supplementary Materials

Table S1. *APOE4* allele frequency in different healthy ethnic populations.

Sample Size	Title 2	Title 3
615	22.70%	Finland [35]
279	20.60%	Sweden [36]
466	17.40%	Denmark [77] [78]
400	14.80%	UK – Scotland [78]
159	13.70%	UK - London [77] [78]
178	12.00%	France – Lille [77] [78]
249	11.90%	France – Reims [78]
303	11.60%	France – Nancy [79]
159	10.70%	France - Strasbourg [77] [78]
171	8.20%	France – Toulouse [77] [78]
173	10.70%	Switzerland-Geneva [78]
100	10.00%	Spain-Barcelona [77] [78]
186	6.10%	Spain – Madrid [77] [78]
352	9.80%	Italy-Padua [77] [78]
195	7.20%	Italy-Rome [54] [78]
280	5.20%	Sardinia [54]
174	6.3%	Sicily [56]
335	7.0%	Cyprus [80]

Table S2. *APOE*4 allele frequency in healthy individuals in various parts of Greece.

Sample Size	Title 2	Title 3
160	13.10%	Northern Greece [41]
555	13.00%	North-western Greece [53]
391	8.58%	Central Greece [38]
216	6.50%	Athens [80]
95	6.60%	Athens-Southern Greece (current study)
